# Antibiotic prescription for HIV-positive patients in primary health care in Mozambique: A cross-sectional study

**DOI:** 10.4102/sajid.v37i1.340

**Published:** 2022-02-28

**Authors:** Candido Faiela, Esperanca Sevene

**Affiliations:** 1 Department of Biological Science, Faculty of Science, Eduardo Mondlane University, Maputo, Mozambique; 2 Department of Physiological Science, Faculty of Medicine, Eduardo Mondlane University, Maputo, Mozambique

**Keywords:** prescription, antibiotics, HIV, primary health care, drug resistance, drug interactions

## Abstract

**Background:**

Antibiotic overuse is a major public health challenge worldwide and it can result in the emergence and spread of drug resistance. In Mozambique, there are limited data related to primary care physicians’ antibiotic prescription patterns. The aim of this study was to assess the antibiotic prescription patterns for HIV- positive patients in primary health care.

**Methods:**

A prospective cross-sectional quantitative study was conducted in eight primary health care units in Southern Mozambique. The study was based on recording outpatient prescriptions using a structured questionnaire. Three hundred and sixty-nine prescriptions and clinical records of HIV-positive patients from 31 prescribers were assessed. A total of eight general practitioners, 13 medical technicians and 10 nurses participated.

**Results:**

Antibiotics were used in 65.9% of prescriptions, with an average of 0.9 antibiotics per prescription. Of a total of 334 prescribed antibiotics, 69.8% were for the treatment of infections and 30.2% for prophylaxis. Penicillin (29.2%), sulphonamides (19.7%), and quinolones (16.3%) were the most prescribed classes of antibiotics for treatment. For prophylaxis, only sulphonamides (93.1%) and macrolides (6.9%) were prescribed. The diagnosis was the only variable that had a significant association with antibiotic prescription (*p* < 0.001). Most of penicillins (68.0%) and sulphonamides (21.4%) were prescribed to treat infections related to the respiratory tract.

**Conclusion:**

The prescription of antibiotics was high and influenced by patient clinical conditions. Antibiotics were prescribed either for treatment or prophylaxis of infections, mostly to treat respiratory tract infections. Prescribers should be encouraged to adopt a rational use of antibiotics to reduce unnecessary prescriptions.

## Introduction

Antibiotics are often prescribed for HIV-positive patients to prevent or treat opportunistic and associated infections. They are sometimes administered in combination with other medications, such as antiretrovirals (ARVs), antivirals, antifungals, antiparasitics, and antidiarrheals.^1^ Simultaneous administration of antibiotics with other medicinal products may result in synergistic or antagonistic drug interactions.^2^ Seden et al. reported an 18.7% prevalence of drug-drug interaction among patients on ARV drugs in Kampala, and the most common interactions were with antibiotics (4.8%).^3^ Schlaeppi et al. found a high prevalence (33.0%) of potential drug-drug interaction in patients on ARVs in Tanzania being more prevalent with antimicrobial drugs.^4^ The potential interaction between clarithromycin and clindamycin with ARVs (nevirapine, efavirenz and ritonavir, saquinavir; respectively) are also described.^4^

Drug interaction may decrease the effectiveness of one drug, contributing to therapeutic failure, but may also cause an increased pharmacological effect leading to the emergence of adverse drug reactions (ADRs). Patients with low CD4 levels are more prone to adverse effects and toxicity resulting from drug therapy,^5^ which may accelerate disease progression and lead to patient death.^6^

Antibiotics are a class of drugs most prescribed and stand out for the higher incidence of adverse reactions.^7^ However, these reactions could be avoided through rational drug use strategies. As a result of overuse and misuse of antibiotics, there is an increasing development of antibiotic resistance by many bacteria worldwide.^8,9,10^ The emergence of resistant strains has been attributed, among other reasons, to the inappropriate use of antibiotics, in conditions that antibiotic therapy is not indicated, especially in viral infections such as influenza and common cold, that can be resolved without treatment.^11^

In Mozambique, there are limited data on physician antibiotic prescription patterns. Most of the available studies focus on antimicrobial drug resistance.^12,13,14,15,16^ Therefore, this study was conducted to assess the antibiotic prescription patterns for HIV-positive patients attending primary healthcare in Maputo and Matola cities.

## Methods

### Study design

A cross-sectional descriptive study was conducted with prospective data collection from the prescriptions and records of HIV-positive patients from March 2013 to September 2013.

### Study site and population

The study was conducted in urban and peri-urban areas of Maputo and Matola cities, located in Southern Mozambique covering an area of 300 km^2^ (in Maputo) and 367 km^2^ (in Matola). In 2013, there were in total 35 primary level health facilities (HFs) in both the cities. Primary health care is the first level of healthcare and is characterised by a set of health actions, at the individual and collective level, which covers health promotion and protection, disease prevention, diagnosis, treatment, rehabilitation, harm reduction, and health maintenance. We selected in total eight primary care HFs according to established criteria, which included the existence of prescribers in the screening and consultation rooms, attendance of more than 600 HIV patients per month, and a pharmacy dispensing medication to HIV patients. All selected HFs had antiretroviral therapy (ART) service, patient counselling service, and voluntary testing service, and appropriate follow-up.

We only targeted HIV-positive patients because HIV weakens the immune system, resulting in opportunistic infections that may require antibiotic use.^1^ With advances in antiretroviral therapy (ART), HIV-positive individuals become well controlled, and the risk of infection reduce substantially and, in most cases, is similar to HIV-negative individuals.^17^ There is a perception that these advances in ART are not accompanied by changes in prescription patterns for this specific group and persist in the high empirical use of antibiotics. Furthermore, there are several strategies directed to this group and we thought targeting HIV-positive patients would add information to the existing strategies towards the use of antibiotics.

The primary care for HIV-positive people in the study area is provided by general practitioners, medical technicians, and nurses. All HIV-positive patients of all ages who presented consecutively at HF medical visit with a complaint were included in the study, based on the following inclusion criteria: (1) HIV-positive patient in follow-up or diagnosed on the same day of consultation, (2) absence of severe pathology that would interfere with the ability to consent and (3) accepting freely to sign the consent.

According to the World Health Organization (WHO), a statistically viable antibiotic prescribing analysis requires a minimum of 100 prescriptions.^18^ Thus, a total of 369 HIV-positive patient records, with and without antibiotic prescription were enrolled for further analysis.

### Data collection

Data collection was based on self-completion of a questionnaire by the prescribers. The questionnaire was structured with questions related to prescriber identification, patient socio-demographic data, signs and symptoms, laboratory tests, diagnosis, and drug prescription. The questionnaires were placed in the screening and consultation rooms of the HF so that the prescriber prospectively filled out whenever he or she treated an HIV-positive patient after the patient gave free informed consent. For cases requiring additional tests, the prescriber withheld the questionnaire to complete with the results of the tests requested. The researchers were responsible for training the prescribers to fill out the questionnaires, monitor the filling, collecting the questionnaires, clean up the data (completeness, incongruous data, unreadable data), and data entry in the database created on the Statistical Package for Social Sciences (SPSS) version 20.

### Data analysis

Data were analysed using SPSS version 20. Data analysis was performed descriptively by drawing up frequency tables. The descriptive analysis was based on the characterisation of the pattern of antibiotic prescription in general, by socio-demographic and clinical features.

Univariate analysis was performed for the following variables: antibiotic prescription, antibiotic class, prescriber gender, patient age, category and time of service. For the age variable, the measures of central tendency and dispersion were also calculated. For bivariate analysis, the Pearson’s chi-squared test with a 95% confidence interval was used to verify if there was an association between patient characteristics (socio-demographic and clinical) and an antibiotic prescription, and *p* ≤ 0.05 were considered statistically significant. For variables (age and clinical diagnosis) where expected frequencies below five were found, Fisher’s exact test^19^ was used.

### Data definitions

Drugs were classified according to the Mozambique National Medicines Formulary (Formulário Nacional de Medicamentos [FNM]) classification following the WHO recommended classification (Anatomical Therapeutic Chemical Classification). Non-FNM drugs were classified as extra-formulary. And the diagnoses or indications for antibiotic use were classified according to the International Classification of Diseases (ICD10).

To calculate the frequency of antibiotic prescriptions, the proportion of medical visits in which at least one antibiotic was prescribed was considered.^11^ For the variable, the class of antibiotics, the classification according to the pharmacological group (Penicillins, aminoglycosides, macrolides, quinolones, sulphonamides, tetracyclines and other antibiotics) was considered.

For the ART variable, two categories were defined: ART Yes and ART No. All patients who were receiving ARV drugs and those who started ART on the day of the medical visit were included in the category of ART Yes. Those patients who were not yet eligible to start ART were included in the category of ART No.

With an antibiotic prescription, all patients who received a prescription with at least one antibiotic for either therapeutic or prophylactic purposes were considered. No prescription antibiotics, for patients who received a prescription for drugs that did not belong to the antibiotic group.

### Ethical considerations

The study was approved by the Mozambique’s National Bioethics Committee for Health with reference number 258/CNBS/12. All methods were carried out by relevant guidelines and regulations. Informed consent was obtained from all participating prescribers and HIV-positive patients.

### Patient and public involvement

This research was performed without patient involvement. Patients were not invited to design or comment on this study and were not consulted to develop outcomes or analyse results. Patients were not invited to contribute to the writing of this manuscript.

## Results

### Participants’ characteristics

Data were collected from 31 prescribers who recorded prescription information on 369 medical visits of HIV-positive patients. Most of the prescribers were women (54.8%), aged 20–35 years (45.1%), medical technicians (41.9%), and with more than 10 years of service time (53.4%) (Table 1).

**TABLE 1:** Prescribers’ demographic characteristics.

Characteristics	*N*	%
**Sex**		
Male	14	45.2
Female	17	54.8
**Age (years)**		
20–35	14	45.1
36–49	10	32.3
50–65	7	22.6
**Category**		
General practitioner	8	25.8
Medical technician	13	41.9
Nurse	10	32.3
**Service time (years)**		
0–10	14	46.6
> 10	17	53.4
**Total**	**31**	**100.0**

For the attendees, the majority were women (63.7%), aged between 25 years and 49 years (74%) with a mean (standard deviation [s.d.]) age of 37 (11.7) years and on ART (71.3%) (Table 2).

**TABLE 2:** Antibiotic prescribing by patient characteristics.

Characteristics	Antibiotic prescribing				Total		*p*
	Yes		No				
	*n*	%	*n*	%	*N*	%
**Sex**							
Male	91	37.1	43	34.7	134	36.3	0.642
Female	154	62.9	81	65.3	235	63.7	
**Age (years)**							
0–14	4	1.6	6	4.8	10	2.7	0.063
15–24	22	9.0	8	6.5	30	8.1	
25–39	124	50.6	63	50.8	187	50.7	
40–49	63	25.7	23	18.5	86	23.3	
50–64	30	12.2	19	15.3	49	13.3	
65+	2	0.8	5	4.0	7	1.9	
**ART**							
Yes	168	68.6	95	76.6	263	71.3	0.107
No	77	31.4	29	23.4	106	28.7	
**HIV stage**							
Stage I (*n* = 124)	88	35.9	36	29.0	124	33.6	0.445
Stage II (*n* = 124)	76	31.0	48	38.7	124	33.6	
Stage III (*n* = 104)	70	28.6	34	27.4	104	28.2	
Stage IV (*n* = 17)	11	4.5	6	4.8	17	4.6	
**Diagnosis**							
Cardiovascular system (I)	3	1.2	22	17.4	25	6.8	< 0.0001
Infectious and parasitic disease (A)	2	0.8	3	2.4	5	1.4	
Genitourinary tract (N)	39	16.0	3	2.4	42	11.4	
Bone-muscular system and connective tissue (M)	6	2.5	4	3.2	10	2.7	
Skin and sub cutis tissue (L)	22	9.1	29	23.0	51	13.8	
Respiratory tract (J)	74	30.5	7	5.6	81	21.8	
General symptoms (R)	16	6.6	23	18.3	39	10.6	
Gastrointestinal tract (K)	37	15.2	23	18.3	60	16.3	
Nervous system (G)	2	0.8	12	9.5	14	3.8	
No information	42	17.3	0	0.0	42	11.4	
**Prescriber Category**							
General practitioner	53	22.0	34	27.0	87	23.6	0.374
Medical technician	107	44.0	58	46.0	165	44.7	
Nurse	83	34.0	34	27.0	117	31.7	
**Service time (years)**							
0–10	119	49.0	53	41.9	172	46.6	0.200
> 10	124	51.0	73	58.1	197	53.4	
**Total**	**243**	**100.0**	**126**	**100.0**	**369**	**100.0**	

ART, antiretroviral therapy.

### Frequency of antibiotics prescription

Antibiotics were prescribed in 65.9% (*n* = 243) of medical visits (Table 2). Most antibiotics were prescribed to female patients (62.9%), adults 25–49 years old (76.3%), HIV diseases stages I and II (66.9%), on ART (68.6%). Children (0–14 years) and the elderly (65+ years) received antibiotics less frequently (1.6% and 0.8%, respectively) compared with young and adults (15–64 years). Prescribers’ category (*p* = 0.374) and length of service (*p* = 0.200) did not significantly influence the prescription of antibiotics (Table 2).

### Number of antibiotics prescribed in each prescription

**FIGURE 1:**
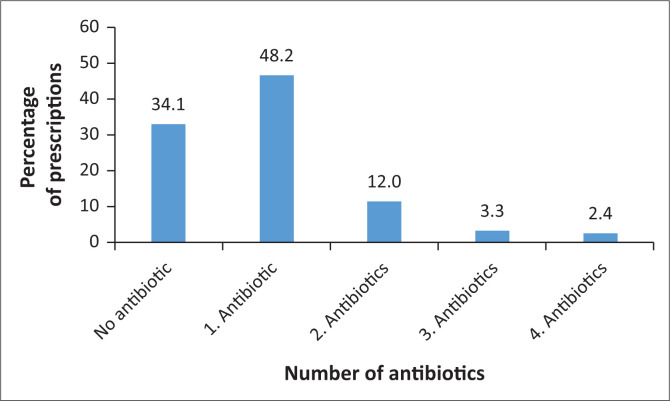
Number of antibiotics prescribed in each prescription.

Among those patient visits with antibiotics prescribed, 48.2% received one antibiotic, 12.0% received two different kinds of antibiotics, 3.3% received three different kinds of antibiotics and 2.4% received four different kinds of antibiotics (Figure 1). Overall, the antibiotic combinations were 17.6%. In the antibiotic combinations, 69.4% were from two different classes, 24.2% of three classes, and 6.4% of four different classes (Table 3). The association between penicillin and sulphonamide was the most frequent with 29.2%, followed by macrolide associated with quinolone and metronidazole (12.3%).

**TABLE 3:** Association of classes of antibiotics.

Number of classes of antibiotics	Association of antibiotics	*n*	%	Total	
				*N*	%
2	Penicillin + Sulphonamide	19	29.2	43	69.4
	Penicillin + Tetracycline†	3	4.6		
	Penicillin + Macrolide	2	3.1		
	Aminoglycoside + Macrolide	1	1.5		
	Aminoglycoside + Metronidazole	1	1.5		
	Aminoglycoside + Tetracycline	1	1.5		
	Macrolide + Quinolone	1	1.5		
	Macrolide + Sulphonamide	5	7.7		
	Macrolide + Metronidazole	1	1.5		
	Quinolone + Metronidazole	1	1.5		
	Quinolone + Sulphonamide	2	3.1		
	Sulphonamide + Metronidazole	6	9.2		
3	Penicillin + Tetracycline + Sulphonamide†	1	1.5	15	24.2
	Penicillin + Sulphonamide + Quinolone	1	1.5		
	Penicillin + Aminoglycoside + Chloramphenicol	1	1.5		
	Aminoglycoside + Tetracycline + Metronidazole	2	3.1		
	Macrolide + Quinolone + Metronidazole	8	12.3		
	Macrolide + Quinolone + Sulphonamide	2	3.1		
4	Aminoglycoside + Tetracycline + Metronidazole + Sulphonamide†	1	1.5	4	6.4
	Macrolide + Quinolone + Sulphonamide + Metronidazole†	3	4.6		

† , Associations not recommended because of increased risk of adverse reactions: Sulphonamide + Metronidazole and Penicillins + Tetracyclines.

### Antibiotics choice for prescription

Antibiotics were prescribed in a total of 334 patients, of which 233 (69.8%) were for treatment and 101 (30.2%) for prophylaxis of infections (Table 4). For treatment, penicillin was the most commonly prescribed class of antibiotics with a frequency of 29.2%, followed by sulphonamides (19.7%) and quinolones (16.3%). In contrast, aminoglycosides and tetracyclines were the least prescribed antibiotics with a frequency of 4.3% and 2.6%, respectively. For prophylaxis, only sulphonamides and macrolides were prescribed, with the first-class being prescribed more frequently (93.1%). Of all prescribed sulphonamides (*n* = 139), 67.1% were used for prophylaxis of infections.

**TABLE 4:** The choice for prescribing a class of antibiotic.

Antibiotic class	Treatment		Prophylaxis		Total	
	*n*	%	*n*	%	*n*	%
Penicillin	68	29.2	0	0.0	68	18.4
Aminoglycoside	6	2.6	0	0.0	6	1.8
Macrolide	31	13.3	8	6.9	39	14.3
Quinolone	38	16.3	0	0.0	38	14.4
Sulphonamide	46	19.7	93	93.1	139	37.9
Tetracycline	10	4.3	0	0.0	10	3.0
Others	34	14.6	0	0.0	34	10.2
**Total**	**233**	**100.0**	**101**	**100.0**	**334**	**100.0**

We assessed the existence of an association between antibiotic prescription and patient socio-demographic and clinical characteristics (Table 2). The diagnosis was the only variable that had a significant association (*p* < 0.0001).

Most of the penicillin (68.0%) prescribed were used to treat respiratory tract infections (Table 5).

**TABLE 5:** Antibiotic prescribing following diagnosis.

Diagnosis (ICD10)	Penicillins		Aminoglycosides		Macrolides		Tetracyclines		Quinolones		Sulphonamides		Other antibiotics	
	*n*	%	*n*	%	*n*	%	*n*	%	*n*	%	*n*	%	*n*	%
Cardiovascular system (I)	0	0.0	0	0.0	0	0.0	0	0.0	0	0.0	3	2.1	0	0.0
Infectious and parasitic diseases (A)	1	1.5	0	0.0	1	2.6	0	0.0	0	0.0	0	0.0	0	0.0
Genitourinary tract (N)	8	11.6	4	66.6	17	44.8	5	50.0	24	64.1	10	7.1	17	50.0
Musculoskeletal system and connective tissue (M)	0	0.0	0	0.0	1	2.6	0	0.0	1	2.6	4	2.8	0	0.0
Skin and subcutaneous tissue (L)	4	5.8	0	0.0	10	26.3	0	0.0	0	0.0	14	10.6	0	0.0
Respiratory tract (J)	46	68	1	16.7	8	21.1	4	40.0	6	15.4	30	21.4	1	3.0
General symptoms (R)	6	8.7	0	0.0	0	0.0	0	0.0	0	0.0	11	7.8	0	0.0
Gastrointestinal tract (K)	2	2.9	0	0.0	1	2.6	1	10.0	7	17.9	25	17.7	15	44.0
Nervous system (G)	1	1.5	1	16.7	0	0.0	0	0.0	0	0.0	1	0.7	1	3.0
Unknown	0	0.0	0	0.0	0	0.0	0	0.0	0	0.0	42	29.8	0	0.0

ICD, International Classification of Diseases.

Amoxicillin and a combination of amoxicillin and clavulanic acid were the most prescribed penicillin (62.3%), followed by phenoxymethyl penicillin (21.7%) and benzyl penicillin (10.1%). Aminoglycosides were specially prescribed to treat genitourinary tract infections (66.6%), with kanamycin being the most prescribed aminoglycoside (85.7%). Macrolides were mainly used to treat infections of the genitourinary tract (44.8%), skin and subcutaneous tissue (26.3%), and respiratory tract (21.1%), with erythromycin (57.9%) being the most commonly prescribed macrolide. Tetracyclines were used to treat genitourinary (50.0%) and respiratory (40.0%) infections, with doxycycline being the most commonly prescribed tetracycline (70.0%).

## Discussion

The study found a considerable use of antibiotics for HIV-positive patients. They were prescribed in 65.9% of all prescriptions with an average of 0.9 antibiotics per prescription. This frequency is high compared to the WHO reference of 20% – 26.8%.^19^ The study recruited only HIV-positive patients. This group is more prone to develop opportunistic infections that can be treated or controlled by antibiotics and it may have influenced the high rate of prescription. The rate is also higher compared to studies reported in Nigeria^20^ (43.3%), Brazil (12.5%^21^ – 56.0%^22^), and Norway^23^ (27.0%). Other studies in different countries also reported higher rates such as Brazil^24^ (66.0%), Tanzania^25^ (84.9%), and Thailand^26^ (81.0%). But none of these studies had HIV-positive patients as the study population. These divergences may reflect the antibiotic use in different settings, as well as a different behavioural pattern of prescribers in those countries.

Among several factors that could influence antibiotic prescribing, only the diagnosis had a statistically significant association (*p* < 0.0001). Respiratory tract diseases were the diagnosis where more antibiotics were prescribed (36.8%), followed by genitourinary tract diseases (19.4%) and gastrointestinal tract (18.4%). Respiratory and gastrointestinal tract infections are common in these patients, and they are the most susceptible to empirical use of antibiotics, especially in low-income countries where access to diagnostic exams is scarce. The most common antibiotics used in these cases are penicillin and sulphonamides.^27,28,29^ With advances in ART, HIV-positive individuals become well controlled, and the risk of infection reduce substantially and, in most cases, is similar to HIV-negative individuals.^18^ There is a perception that these advances in ART are not accompanied by changes in prescription patterns for this specific group and persist in the high empirical use of antibiotics.

The higher prevalence of antibiotic prescriptions for respiratory tract diseases, observed in this study, is consistent with what was reported in other countries.^20,30^ There is evidence of irrational use of antibiotics for the treatment of respiratory system diseases,^11,20,21,22^ especially upper respiratory tract diseases (sinusitis, influenza, and simple cold).^31,32,33^ Most respiratory infections are of viral aetiology,^33,34,35^ and the use of antibiotics is not indicated, being restricted to patients with a confirmed diagnosis of a bacterial infection or with high suspicion and when prophylaxis is strongly recommended^36^ when the consequences of infection can be severe. However, this study was conducted in winter, where there is an increase in cases of upper respiratory tract infections, which may have contributed to increased rates of antibiotic prescribing.

Some patients who were not eligible for antibiotic therapy received a prescription for this class of drugs. Therefore, antibiotics were not expected to be prescribed to individuals whose diagnoses were related to non-infectious diseases of the cardiovascular system and nervous system.

For the treatment of infections, penicillin and sulphonamides were more commonly prescribed. Several studies report higher consumption of penicillins in primary healthcare units^11,18,20,24,^ and hospitals.^37^ The predominance of penicillin use aligns with practice in other countries.^20,27^ In this study, more than half of the penicillins were used for the treatment of respiratory tract infections. Amoxicillin and a combination of amoxicillin and clavulanic acid were the most prescribed penicillin. According to Paganotti,^24^ amoxicillin is the antibiotic of the first choice against the main bacterial agents that cause respiratory infections. Some studies indicate that amoxicillin is the most prescribed antibiotic to treat bacterial infections in vulnerable groups, such as children and patients with compromised immunity, either in primary care or in emergency services.^22,24^ Overuse of penicillin may result in bacterial resistance,^18^ as observed in a study conducted in Cambodia, where 96.2% of HIV-positive patients had resistance to amoxicillin.^38^ In Mozambique, resistance to penicillin has been reported in 44% of the strains causing pneumococcal pneumonia in patients, irrespective of their HIV status.^39^

High consumption of penicillin has been observed in health units providing primary health care. Penicillins are preferred drugs in almost all infections, except for urinary tract infections, because of their inadequate pharmacokinetic characteristics, being quinolones, antibiotics most suitable for this condition. Quinolones are not the first line of treatment for urinary tract infections in Mozambique, but given their pharmacological characteristics,^40^ their use is beginning to be high, which may compromise the potential of this group of drugs and the emergence of resistance.^41^ Therefore, more than half of the prescribed quinolones in this study were used to treat genitourinary tract infections, although the literature also describes their successful therapeutic use for respiratory tract infections.^41,42,43^ This study shows that in addition to genitourinary infections, quinolones were also used to treat gastrointestinal and respiratory infections.

Most of the sulphonamides were prescribed for the treatment of respiratory and gastrointestinal infections as well as for the prophylaxis of infections mainly in undiagnosed patients. Cotrimoxazole is indicated as an alternative to ß-lactam allergic patients in the urogenital tract and respiratory tract infections. It is also the first choice for the treatment and prophylaxis of *P. carinii* pneumonia in patients with immunosuppression.^40^ The predominant use of sulphonamides in this study may be related to the fact that it is part of the WHO recommendations for the reduction of morbidity and mortality by opportunistic diseases associated with HIV or AIDS. The WHO recommends prescribing cotrimoxazole prophylactically to all HIV-positive patients in stages 2, 3, and 4 in resource-limited settings, such as the lack of laboratories for CD4 cell counting and viral load measurement.^44^ Nearly all health units included in this study lack these facilities. Therefore, in this setting, it is recommended to prescribe cotrimoxazole to all patients in these stages of the disease. We found that more than half of the prescribed cotrimoxazole was for patients in stages 2–4. The reduction of morbidity and mortality because of opportunistic diseases in HIV-positive patients taking co-trimoxazole has been reported.^36,44^ However, the problem is inappropriate use, which contributes to increased bacterial resistance.^9^

Studies in Mozambique reported high levels of co-trimoxazole resistance related to the past inappropriate use of sulphonamides for the treatment of malaria (sulfadoxine/pyrimethamine) and prophylaxis against *P. carinii* pneumonia in HIV-positive patients.^14,16^

The study relied on prescriber self-reported practices. Therefore, there may have been biases towards prescribing behaviours and Hawthorne effect because of prescribers’ awareness of being observed. The study was conducted in a primary healthcare setting where access to accurate diagnosis was limited because of lack of laboratory support. We did not measure antibiotic use and prescription audit to determine the appropriate use of antibiotics. Despite these limitations, our study provides a good insight into the antibiotic prescription patterns for HIV-positive patients in primary health care in Mozambique. Data from this study can be used to enhance medical education, antibiotic surveillance, and prescribing patterns in our settings.

## Conclusion

The prescription of antibiotics for HIV-positive patients in the study area was high and influenced by patient clinical conditions. Antibiotics were prescribed either for treatment or prophylaxis of infections and in some cases, associations of different classes were used. Penicillin and sulphonamide were the most prescribed antibiotics for the treatment of infections. Most of these antibiotics were indicated to treat respiratory tract infections. We recommend a more detailed study to measure the appropriate use of antibiotics. Actions need to be taken to encourage healthcare professionals to adopt a rational use of antibiotics, to reduce unnecessary prescriptions, especially in primary care units attended by HIV-positive patients.
